# Effects of COVID‐19 lockdown on lifestyle behaviors in children with obesity: Longitudinal study update

**DOI:** 10.1002/osp4.581

**Published:** 2021-12-27

**Authors:** Angelo Pietrobelli, Nicole Fearnbach, Alessandro Ferruzzi, Massimiliano Vrech, Moonseong Heo, Myles Faith, Luca Pecoraro, Thomas Zoller, Franco Antoniazzi, Giorgio Piacentini, Steven B. Heymsfield

**Affiliations:** ^1^ Department of Surgical Science, Dentistry, Gynecology and Pediatrics Pediatric Unit Verona University Medical School Verona Italy; ^2^ Pennington Biomedical Research Center LSU System Baton Rouge Louisiana USA; ^3^ Department of Public Health Sciences Clemson University Clemson South Carolina USA; ^4^ Graduate School of Education Department of Counseling, School and Educational Psychology University at Buffalo‐State University of New York Buffalo New York USA

**Keywords:** diet, homebound, overweight, physical activity, school program

## Abstract

**Objective:**

A previous report from our group identified directionally unfavorable dietary and lifestyle behavior trends in longitudinally monitored children and adolescents with obesity early in the COVID‐19 pandemic lockdown. The current study aimed at extending these previous observations in youths with obesity on the dietary and lifestyle behavioral consequences of the extended COVID‐19 lockdown in Verona, Italy.

**Methods:**

The sample included 32 children and adolescents with obesity participating in the longitudinal OBELIX study. Diet and lifestyle information were collected pre‐pandemic, 3 weeks into the national lockdown, and 9 months later when home confinement continued to be mandatory. Changes in outcomes over the study time points were evaluated for significance using repeated‐measures ANOVA and post‐hoc pairwise *t*‐tests with Bonferroni corrections.

**Results:**

As previously reported, meals/day, fried potato intake, and red meat ingestion increased significantly (*p* < 0.001) during the initial lockdown. Sleep time and screen time increased and sports participation decreased significantly (*p* < 0.001) during the initial lockdown. These changes in health behaviors remained significantly different from baseline at the second lockdown assessment, with the exception sleep time returned to baseline levels.

**Conclusions:**

Unfavorable diet and lifestyle behavioral changes in response to the initial COVID‐19 lockdown in children and adolescents with obesity have largely been sustained over the course of the pandemic. There is an urgent need to intervene on these behaviors to prevent further deleterious effects on long‐term child health; access to weight management care is critically important for these children. In addition to intervening on these behaviors, our findings should help to inform ongoing lockdown policies.

## 1 INTRODUCTION

The coronavirus disease 2019 (COVID‐19) pandemic has continued to have profound social, health, and economic consequences. Among these untoward effects is the abrupt closure of in‐class school programs for children and adolescents, who by necessity must remain in their homes during the lockdown as a means of limiting COVID‐19 transmission. In an earlier report from a longitudinal cohort study, we found that house‐bound children and adolescents with obesity living in Verona, Italy displayed worsening of their diet and lifestyle behaviors including sleep time and activity levels.^1^ The recent retrospective cohort study of Woolford and colleagues,^2^ using Kaiser Permanente Southern California electronic health record data, supports and extends these observations. The author's reported that children between the ages of 5 and 17 years gained a significant amount of weight during the lockdown. Similarly, Lange et al.^3^ found in a sample of over 400,000 American children and adolescents the rate of increase in BMI almost doubled during the pandemic compared to pre‐COVID levels. The original assumption regarding the lockdown in Italy was that it would be a transient period that would bring the pandemic under control. However, the emergence of new, more virulent strains of COVID‐19 necessitated repeated and prolonged home confinement periods. These unexpected events led us to survey again the same group of children and adolescents with obesity with the aim of establishing if the untoward changes in weight‐related behaviors persisted beyond our initial observations early in the pandemic.

## 2 METHODS

### 2.1 Study design

Children and adolescents with overweight or obesity (BMI >25 kg/m^2^) were participants in the longitudinal observational OBELIX study. The original reported study^1^ included two time points with 41 participants, a baseline evaluation prior to the pandemic (13 May to 30 July 2019) and 3 weeks following implementation of the mandatory lockdown (10 March 2020) in Verona, Italy. A second identical follow‐up telephone interview was conducted (10 January to 30 January 2021) in 32 of the original participants who agreed to be interviewed when the mandatory lockdown was extended beginning in April 2020 and ended in February 2021. All children examined during this period were required to participate in an additional lockdown with restrictions that varied over several months. The children under the age of 13 years attended school from September 2020 onward following their summer break from June to September 2020. Boys and girls over the age of 13 years attended distance learning activities from September 2020 onward. A structured lifestyle program was not administered to the children and adolescents during the shelter‐in‐place periods.

## 3 BEHAVIORAL QUESTIONNAIRE

The data collection instrument consisted of 12 questions about dietary patterns (e.g., portions of red meat, potato chips, etc.) and sleep, sports participation, and screen watching. A meal was defined as a structured, nonliquid ingestive event, including breakfast, lunch, afternoon snacks, and dinner. The investigator conducted 10‐min telephone interviews with the parents of each participant. Time spent in sports prior to and during the lockdown were defined as any physical activity (e.g., jogging, playing in the backyard, etc.) as it was not possible to participate in organized sports such as soccer, swimming, volleyball, and basketball. Some educational programs were broadcasted during the lockdown, although the screen time question related specifically to nonschool activities. The current study was approved by the Hospital Institutional Review Board (Protocol: 5384, 01/29/2019). All parents and children provided informed consent.

### 3.1 Statistical methods

Descriptive statistics of the participants' baseline characteristics are presented as mean and standard deviation (SD) for continuous variables and frequency and percentages for categorical variables. Three comparisons were made (evaluations 1 [T1] and 2 [T2] vs. baseline [T0] and evaluation 2 vs. evaluation 1) using a repeated measure one‐way analysis of variance (ANOVA) and post‐hoc pairwise *t*‐tests with Bonferroni *p*‐value corrections. All statistical analyses were performed using SPSS (IBM SPSS Statistics, version 25).

## 4 RESULTS

### 4.1 Participants

The 32 participants included 16 males and 16 females with a mean baseline age of 12.8 ± 3.0 (range 7–17) years (Table 1). Of these participants, 17 were under the age of 13 years (7 males and 13 females) and 15 were over the age of 13 years (9 males and 6 females). Baseline BMI was similar at about 30 kg/m^2^ in males and females. Baseline reported activities included running/soccer (22%), swimming (22%), jogging (14%), volleyball/basketball (13%), boxing/martial arts (9%), and dancing (13%); only 6% reported no sports activities.

**TABLE 1:** Participant baseline characteristics

	Females (*N* = 16)		Males (*N* = 16)		Total (*N* = 32)	
Characteristics	Mean ± SD	Range	Mean ± SD	Range	Mean ± SD	Range
Age (years)	12.5 ± 2.9	(6.0, 17.0)	13.1 ± 3.1	(7.0, 18.0)	12.8 ± 3.0	(6.0, 18.0)
Height (cm)	155 ± 12.9	(124, 186)	159.1 ± 18.4	(126, 168)	157.1 ± 15.7	(124, 186)
Weight (kg)	73.3 ± 16.3	(40, 117)	76.5 ± 23.1	(40, 99.5)	74.9 ± 19.7	(40, 117)
BMI (kg/m^2^)	30.1 ± 3.9	(23.1, 37.3)	29.4 ± 3.9	(25.2, 39.9)	29.8 ± 3.8	(23.1, 39.9)
WC (cm)	87.7 ± 10.3	(77, 108)	91.0 ± 11.7	(69, 110)	89.4 ± 11.0	(69, 1100)

Abbreviations: BMI, body mass index; WC, waist circumference.

### 4.2 Questionnaire observations

The health behavior questionnaire findings are presented in Table 2. There were six questions that examined dietary behaviors. The question on meals/day showed a significant (*p* < 0.01) main effect of time, with an increase from T0 (4.28 ± 0.89) to T1 (5.15 ± 1.12), but no further change at T2 (5.47 ± 0.92). With respect to meal quality, the questions on vegetable servings/day and fruit servings/day showed no time effects. By contrast, servings of fried potatoes and red meat showed significant (*p* < 0.001) main effects of time, with an increase from T0 (0.08 ± 0.26) to T1 0.64 ± 0.78), but no further changes at T2 (0.58 ± 0.71). There was no effect of time for sugary drinks/day.

**TABLE 2:** Results of the questionnaire survey^a^

Variable	Baseline	Lockdown	Lockdown	Δ_1_	Δ_2_	Δ_3_	Significance
	T0	T1	T2	Τ1−Τ0	Τ2−Τ0	Τ2−Τ1	
Meals (#/day)	4.28 ± 0.89	5.15 ± 1.12	5.47 ± 0.92	0.88 ± 1.48	1.19 ± 1.20	0.31 ± 1.47	A
Vegetable intake^b^	1.36 ± 0.76	1.23 ± 0.72	1.25 ± 0.60	−0.13 ± 0.64	−0.11 ± 1.06	0.02 ± 0.95	B
Fruit intake^b^	1.16 ± 0.71	1.39 ± 0.70	1.42 ± 0.69	0.22 ± 0.72	0.27 ± 1.07	0.05 ± 1.02	B
Fried Potatoes^b^	0.08 ± 0.26	0.64 ± 0.78	0.58 ± 0.71	0.55 ± 0.82	0.50 ± 0.71	−0.05 ± 1.08	A
Red meat^b^	1.47 ± 0.91	3.16 ± 1.99	3.28 ± 2.08	1.69 ± 2.23	1.81 ± 2.37	0.13 ± 2.03	A
Sugary drinks (#/day)	0.41 ± 0.94	0.81 ± 1.10	0.72 ± 0.73	0.41 ± 1.07	0.31 ± 1.26	−0.09 ± 1.53	B
Screen time (h/day)	2.69 ± 1.69	7.52 ± 2.24	7.58 ± 2.75	4.94 ± 2.38	4.84 ± 2.86	−0.09 ± 2.80	A
Sleep time (h/day)	8.42 ± 0.89	9.08 ± 1.08	8.41 ± 0.87	0.66 ± 1.33	−0.02 ± 1.25	−0.67 ± 1.42	C
Sports (h/week)	3.36 ± 3.76	1.16 ± 1.33	0.98 ± 1.17	−2.20 ± 3.89	−2.38 ± 4.02	−0.17 ± 1.41	A

^a^ *N* = 32, *X* ± SD.

^b^ Units are serving per day. T0, T1, and T2 are baseline, time 1, and time 2 evaluations. A, significant main effect of time from T0 to T1, but no further change at T2; B, no main time effect; C, significant main effect of time from T0 to T1, but a return to baseline at T2.

Three questions examined lifestyle behaviors. Screen watching showed a significant (*p* < 0.001) main effect of time with an increase from T0 (2.69 ± 1.69) to T1 (7.52 ± 2.24), but no further change at T2 (7.58 ± 2.75). Significant (*p* < 0.008) main effects of time were present for hours of sleep/night with an increase from T0 (8.42 ± 0.89) to T1 (9.08 ± 1.08) and a return to baseline levels at T2 (8.41 ± 0.87). Lastly, a significant (*p* < 0.001) main effect of time was present for sports/week with a decline from T0 (3.36 ± 3.76) to T1 (1.16 ± 1.33), but no further change at T2 (0.98 ± 1.17).

## 5 DISCUSSION

The current longitudinal study of children and adolescents with obesity was fortuitously started as part of an unrelated project taking place several months before the COVID‐19 pandemic unfolded in Europe. Once the lockdown was in place, we were able to again query the parents of our participants using the same dietary and behavioral instruments that were used in the baseline study. Our findings, derived from the third evaluation of the cohort as they remained confined at home, revealed that the unfavorable diet and lifestyle behavioral changes in response to the initial COVID‐19 lockdown have largely been sustained over the course of the pandemic.^1^

Specifically, three dietary measures, meals/day, fried potato intake, and red meat ingestion, increased significantly during the initial lockdown. Both sleep time and screen time increased and sports participation decreased significantly (*p* < 0.001) during the initial lockdown. All six of these changes in health behaviors remained significantly different from baseline at the second lockdown assessment in 2021, with the only exception being sleep time, which returned to baseline levels. Consistent with our findings, European studies conducted in France,^4^ Catalonia,^5^ Italy,^6^ and Greece^7^ reported adverse lockdown effects on physical activity, sleep patterns, and other diet and lifestyle measures in children and adolescents.

Strong evidence suggests that the changes in diet and lifestyle behaviors brought about by the pandemic translate to disproportionate increases in body weight among children and adolescents. In a recent study of almost 200,000 racially and ethnically diverse children and adolescents living in the United States, Woolford et al.^2^ reported that overweight and obesity increased during the pandemic. The absolute increase in percent overweight and obesity among 5‐ through 11‐year‐old was 8.7%, greater than among 12 to 15 and 16‐ to 17‐year‐old adolescents (5.2% and 3.1%), respectively. In another recent study, Lange et al.^3^ evaluated the BMI increase during the pandemic of over 400,000 American children and adolescents between the ages of 2 and 19 years. Compared to pre‐pandemic levels, the rate of increase in BMI almost doubled with the largest increases present in those with pre‐pandemic overweight or obesity. Notably, children ages 5–11 years experienced the greatest acceleration in weight trajectories. Jenssen et al.^8^ evaluated obesity rates in a large and diverse US sample of children and adolescents. The authors found that preexisting disparities in obesity rates widened during the pandemic in relation to race/ethnicity, insurance coverage, and neighborhood socioeconomic status. The lockdown effects on diet and other lifestyle behaviors thus clearly translate to excess weight gain in children and adolescents, particularly among selected age and racial/ethnic groups. Among the likely factors favoring a positive energy balance in the children and adolescents we evaluated is the increase in screen time by about 4 to 5 h/day that was sustained across both of our lockdown evaluations. Additional screen time during the lockdown was a likely substitution for previous physical activity habits, as we saw a concomitant reduction in sports participation during the lockdown to just one‐third of initial levels. Similarly, more in‐home time likely led to significantly more meals eaten per day along with greater amounts of red meat and fried potatoes consumed. In‐home confinement thus creates an unhealthy dynamic for children and adolescents, particularly those with obesity.

The current study has several limitations, including that data was acquired in a small sample from the parents of children and adolescents with obesity. Additionally, we did not have quantitative measures including weight, height, and activity levels at either of the lockdown time points, so our inference is limited to the self‐reported behavioral changes.

In conclusion, our study again affirms the worsening of dietary and behavioral patterns in children and adolescents with obesity who were confined to their homes during the COVID‐19 pandemic. These observations are concordant with the striking increase in overweight and obesity among persons in the 2‐ to 19‐year‐old age group now reported in several studies.^2^, ^3^ School closures, more non‐educational screen time, poor diet, and fewer opportunities for physical activity all likely contribute to this adverse health trajectory. There is an urgent need to intervene on these behaviors to prevent further deleterious effects on child health; access to weight management care is critically important for these children. In addition to intervening on these behaviors, our findings should help to inform ongoing lockdown policies.

## CONFLICT OF INTEREST

The authors declared no conflict of interest.

## AUTHORS CONTRIBUTIONS

Angelo Pietrobelli, Luca Pecoraro, Thomas Zoller, Franco Antoniazzi, and Giorgio Piacentini designed the research; Angelo Pietrobelli, Alessandro Ferruzzi, Massimiliano Vrech, Luca Pecoraro, Thomas Zoller, Franco Antoniazzi, and Giorgio Piacentini conducted the research; Angelo Pietrobelli, Nicole Fearnbach, Moonseong Heo, and Steven B. Heymsfield analyzed data; Angelo Pietrobelli, Nicole Fearnbach, Luca Pecoraro, Alessandro Ferruzzi, Moonseong Heo, Myles Faith, Thomas Zoller, Giorgio Piacentini, and Steven B. Heymsfield wrote paper; Angelo Pietrobelli, Nicole Fearnbach, Luca Pecoraro, Alessandro Ferruzzi, Moonseong Heo, Myles Faith, Thomas Zoller, Giorgio Piacentini, and Steven B. Heymsfield wrote paper had primary responsibility for final content.
